# Expression of cyclin D1, D3, E, and p27 in human renal cell carcinoma analysed by tissue microarray

**DOI:** 10.1038/sj.bjc.6600922

**Published:** 2003-04-29

**Authors:** Y Hedberg, B Ljungberg, G Roos, G Landberg

**Affiliations:** 1 Department of Medical Biosciences, Pathology, Umeå University, Umeå, Sweden; 2 Department of Surgical and Perioperative Sciences, Urology and Andrology, Umeå University, Umeå, Sweden; 3 Division of Pathology, Department of Laboratory Medicine, Lund University, Malmö University Hospital, S-205 02 Malmö, Sweden

**Keywords:** cell cycle, Gl/S transition, cyclin, tissue microarray, protein, renal cell carcinoma

## Abstract

Aberrations in the G1/S transition of the cell cycle have been observed in many malignancies and seem to be critical in the transformation process. Few studies have delineated the presence of G1/S regulatory defects and their clinical relevance in renal cell carcinoma (RCC). Therefore, we have examined the protein contents of cyclin D1, D3, E, and p27 in 218 RCCs, using tissue microarray and immunohistochemistry. The results from a subset of tumours were confirmed by Western blotting and immunohistochemical staining of regular tissue sections. Interestingly, low protein contents of cyclin D1 and p27 were associated with high nuclear grade, large tumour size, and poor prognosis for patients with conventional tumours. We further observed substantial differences in the pattern of G1/S regulatory defects between the different RCC subtypes. The majority of both conventional and papillary cases expressed p27; however, chromophobe tumours generally lacked p27 staining. In addition, conventional RCCs often expressed high cyclin D1 protein levels, while papillary RCCs exhibited high cyclin E. In summary, we have shown that G1/S regulatory defects are present in RCC and are associated with clinico-pathological parameters. The pattern of cell cycle regulatory defects also differed between RCC subtypes.

The G1/S transition is a crucial checkpoint in the control of proliferation during cell cycle progression. With an intact G1/S checkpoint, cells with minor DNA damages are stopped and aberrations are repaired before S-phase entrance. Cells with widespread alterations undergo apoptosis (Harbour and Dean, 2000). Different defects affecting the regulation of the G1/S transition have been detected in various malignancies (Bartkova *et al*, 1996; Gillet *et al*, 1996; Hommura *et al*, 2000; Kawauchi *et al*, 2001). This suggests that cell cycle aberrations probably represent one of a limited number of key events in the transformation process (Hahn *et al*, 1999). Therefore, characterisation of G1/S regulatory proteins and the delineation of different aberrations in tumours are important for the understanding of tumour development and progression.

Several proteins control the G1/S transition, but the activators cyclin D and E are rate limiting for S-phase entrance. The cyclins belong to a large, evolutionary conserved protein family and are expressed at specific cell cycle phases. The cyclin-dependent kinases (cdks) are activated by the cyclins and inhibited by cdk inhibitors such as p16, p21, and p27 (Lees, 1995; Reed, 1997). Cyclin D-activated kinases (CDK4/6) initiate a cascade of events starting with site-specific phosphorylation of pRb, which generates a partial release of the transcription factor E2F. This results in a subsequent activation of E2F-responsive genes such as cyclin E, which enables the formation of active cyclin E/cdk2 complex and an extensive phosphorylation of pRb and S-phase entrance. During the activation of the cyclin E/cdk2 complex, the inhibitor p27 releases from the complex and is sequestered by cyclin D1 (Sherr, 2000).

So far, only a few studies have evaluated cell cycle alterations in human renal cell carcinoma (RCC). Earlier, we have shown that the cyclin D1 and E proteins were highly expressed in a large fraction of RCCs, and high cyclin D3 levels were observed in 16% of the tumours. For most RCCs, the p27 protein content seemed to be unaltered, but in a small fraction of the tumours p27 protein was low or undetectable. Low p27 was associated with poor prognosis and large tumour size in conventional RCCs (Hedberg *et al*, 1999, Hedberg *et al*, 2002a, 2002b). We have also shown that low cyclin D1 protein content was associated with a shorter survival time (Hedberg *et al*, 1999). In contrast, Aaltomaa *et al* (1999) found that the cyclin D1 or p21 protein contents had no prognostic value in RCC. Molecular alterations of the Rb gene and aberrant pRb expression seem to be infrequent in RCC (Presti *et al*, 1996; Lai *et al*, 1997). This suggests that mainly the regulating molecules in the G1/S transition are deregulated in RCC, whereas the substrate pRb is unaffected.

Tissue microarray (TMA) is a new method that enables analysis of a large number of samples using various methods. Selected areas of archival paraffin-embedded tissues are biopsied and mounted in a TMA block, which permits multiple analysis of a large collection of samples and use of a limited amount of tissue. In addition, this method creates a uniform platform to stain and evaluate protein, RNA, and DNA using immunohistochemistry and *in situ* hybridisation methods (Kononen *et al*, 1998; Moch *et al*, 1999; Kallioniemi *et al*, 2001).

This study evaluates the expression pattern of cell cycle proteins involved in the G1/S transition in a large amount of RCC, using the TMA technique and immunohistochemistry. In order to validate the results obtained by the TMA technique, the data were compared with regular immunohistochemistry sections and Western blotting using protein extracts prepared from a subset of the tumours.

## MATERIAL AND METHODS

### Patient data

The TMA blocks contained 238 RCCs, all of which were represented by two tissue cores. Eight tumours were excluded because of necrosis, and 12 tumours were excluded because of disagreement between the two tissue cores considering staining (*n*=7) or morphology (*n*=5). In total, 218 tumours were included and characterised in this study. There were 127 men and 91 women and the mean age of the patients was 65.1 years (range 25–87 years). The samples were collected between 1982 and 1997 at the University Hospital in Umeå, Sweden. All tumour samples were obtained after permission from the patients and during the last 6 years with informed and signed consent. The patients were followed according to a scheduled followup programme including clinical and radiological examinations. At the last followup, 63 patients were alive, 105 patients had died of the disease, and 50 patients were deceased because of other events. The median followup time for patients alive was 97 months (range 38–209 months). Clinical staging was performed according to the 1997 TNM classification system (Sobin and Wittekind, 1997), and nuclear grading was performed according to Skinner *et al* (1971). The tumours were also classified according to the Heidelberg classification system (Kovacs *et al*, 1997). There were 175 conventional, 29 papillary, and 14 chromophobe RCCs. Tumour size was measured on the surgical specimens and/or on computerised tomographies.

### Construction of TMA blocks

One pathologist (GL) screened sections from a large amount of RCC material and selected areas of the paraffin-embedded tissue specimens that contained representative tumour cells. If heterogeneity (regarding differentiation) was observed, then the parts with the lowest differentiation grade were selected. Two tissue cores (0.6 mm in diameter) were taken from each tumour sample and placed in a new recipient paraffin block using a commercially available microarray instrument (Beecher Instruments, USA). Each TMA block included 98 tissue cores and the total RCC material was mounted in six blocks.

### Antibodies and staining procedures

Paraffin sections (4 *μ*m) from the TMA blocks were deparaffinised and microwave treated according to standard procedures before being processed in an automatic immunohistochemical staining machine (Ventana 320-202, Ventana Inc., Tucson). An AEC detection kit was used. Cyclin D1 was detected by monoclonal antibody M7155 (DAKO, Sweden), diluted 1/20, and cyclin D3 by the 14781C antibody (Becton Dickinson, Belgium), diluted 1/50. Antigen retrieval was performed in 0.01 M citrate buffer (pH 6.0) for 15 min. Cyclin E was detected by HE-12 monoclonal antibodies (Santa Cruz, CA, USA), diluted 1/50 after antigen retrieval in EDTA (pH 8.0), and p27 was detected by Kip1 antibody (Transductional Laboratories, KY, USA), diluted 1/200, after antigen retrieval in a citrate buffer (pH 6.0). The Ab-3 antibody (Neomarkers, England) was used for the VEGF staining (1/50) after antigen retrieval in EDTA buffer. Stainings were performed on consecutive sections.

### Evaluation of the immunohistochemical staining

The expression of cyclin D1, D3, E, and p27 was evaluated in four groups (1–4). Tumours that lacked nuclear staining were defined as group 1 and tumours with less than five positive cells per tissue core were defined as group 2. These two groups were defined as tumours with low protein content. The staining pattern of the various proteins in the RCCs with high protein levels varied, and therefore separate definitions for groups 3 and 4 were used. For cyclin D1, tumours with more than five positive cells per tissue core showed nuclear staining in most cells, but with a clear variation in staining intensity. Group 3 was defined as more than five positive cells, but with low cyclin D1 staining intensity, whereas tumours with high intensity were defined as group 4. For cyclin D3, tumours with more than five positive cells per tissue core but less than 80% positive cells were defined as group 3. Group 4 had more than 80% cyclin D3-positive tumour cells. For cyclin E, group 3 was defined as tumours with more than five positive cells per tissue core and less than 50% positive tumour cells, and group 4 was defined as tumours with more than 50% positive cells. For p27, tumours with more than five positive cells per tissue core and a variable staining intensity in the separate cells were defined as group 3 tumours, and those with a high-intensity staining in all tumour cells were defined as group 4. Sixteen of the 218 RCCs lacked nuclear staining of cyclin D1, D3, E, and p27 in the tumour cells. To evaluate whether a fixation error had produced false-negative results, VEGF staining was used as a positive control. Fifteen of the 16 tumours showed a positive VEGF staining and most tumours had positive p27 staining in normal cells and/or cytoplasmic cyclin staining. Therefore, the 16 tumours that lacked nuclear staining of cyclin D1, D3, E, and p27 were included in the analysis.

### Western blotting

Preparation of protein extracts from fresh frozen tissues and electrophoresis were performed as described earlier (Hedberg *et al*, 1999, Hedberg *et al*, 2002a, 2002b). Briefly, protein extract from each sample and a positive control was separated on SDS–polyacrylamide gels and the proteins were transferred to nitrocellulose membranes (Hybond-N, Amersham Int., England). The membranes were probed with anti-cyclin D1 DCS-6 antibodies (1 : 500, a kind gift from Dr Jiri Bartek, Copenhagen, Denmark), anti-HE12 antibodies (1 : 1500, Santa Cruz, CA, USA), anti-cyclin D3 G107-565 antibodies (1 : 250, Pharmingen, San Diego, CA, USA), and actin antibodies (1 : 2000, Boehringer-Mannheim GmbH, Germany). Ponceau Red and actin were used as loading and degradation controls.

Several cyclin E reactive bands were observed in the Western blots, as also described in detail earlier (Keyomarsi *et al*, 1995). Previous studies have shown good agreement between immunohistochemistry and Western blot analyses using HE12, cyclin E antibodies (Nielsen *et al*, 1998) validating the specificity of the antibody.

### Statistical analysis

When both parameters contained nominal data, Fisher's exact test was performed. The Kruskal–Wallis test was used to compare the distributions of a variable for groups. For survival analysis, log-rank tests and Kaplan–Meier curves were used, and for multivariate analysis, the Cox-regression model was used. If no event occurred, the patient was censored at the time of the last clinical followup or death from other causes. A *P*-value less than 0.05 was considered statistically significant. Statistical analysis was performed using the SPSS 9.0 software.

## RESULTS

### Protein expression during G1/S transition in RCC

**Figure 1:**
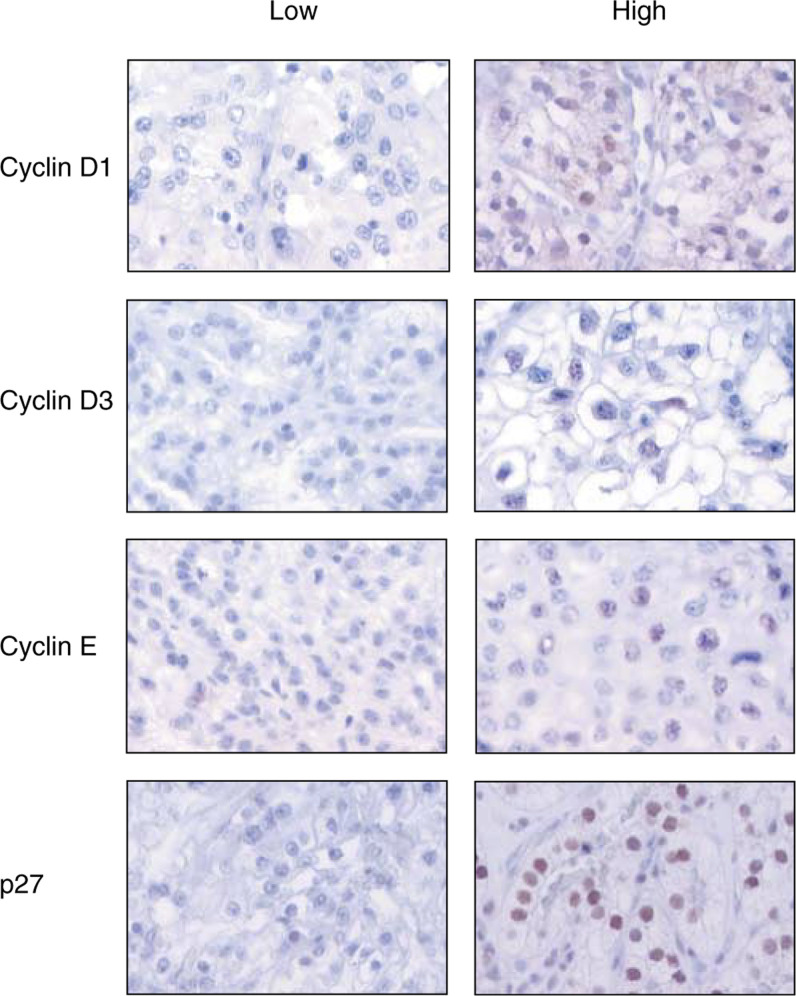
Illustration of cyclin D1, D3, E, and p27 immunohistochemistry staining of RCC using TMA technique. The left column illustrates a low protein expression, and the right column shows tumours with high content of the cell-cycle-regulating proteins.

Examples of immunohistochemistry staining of cyclin D1, D3, E, and p27 are shown in Figure 1

and data are summarised in Table 1

**Table 1:** Protein expression of cyclin D1, D3, E, and p27 evaluated by TMA, graded into four groups from negative (1) to positive (4)

		**Conventional**		**Papillary**		**Chromophobe**	
*Cyclin D1*							
1	Low	64	47%	21	90%	11	79%
2		18		5		0	
3	High	30	53%	2	10%	1	21%
4		62		1		2	

*Cyclin D3*							
1	Low	108	83%	21	90%	10	86%
2		33		5		2	
3	High	17	17%	1	10%	1	14%
4		12		2		1	

*Cyclin E*							
1	Low	72	68%	9	67%	5	46%
2		47		9		1	
3	High	41	32%	7	33%	2	54%
4		14		2		5	

*p27*							
1	Low	16	20%	6	34%	8	64%
2		19		4		1	
3	High	115	80%	18	66%	5	36%
4		21		1		0	

Numbers of cases in each group are noted

. The formalin-fixed tumour blocks had been stored for 2–18 years before the TMAs were constructed. In order to test if the results differed depending on the storage time, the proportion of positively stained tumour cells was plotted for each year (data not shown). The results were uniform during the study period, indicating that the studied proteins were stable at least up to 18 years.

### Evaluation of the TMA technique

**Figure 2:**
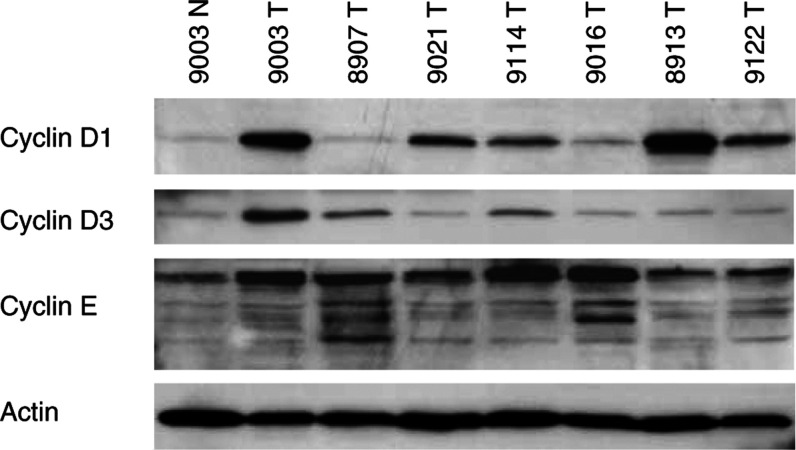
Illustration of Western blot analysis of cyclin D1, D3, E, and actin in RCC. Actin was used as a loading control. The first lane, marked 9003 N, represents corresponding kidney cortex from patient 9003, whereas the tumour sample was loaded in lane 2. The other six lanes contain tumour samples to illustrate the variation in protein contents between the tumours.

The relative protein concentrations of cyclin D1, D3, and E have been previously analysed by Western blotting in 68 of the tumours included in the TMA (Hedberg *et al*, 1999, Hedberg *et al*, 2002a, 2002b). Representative Western blots are shown in Figure 2

**Figure 3:**
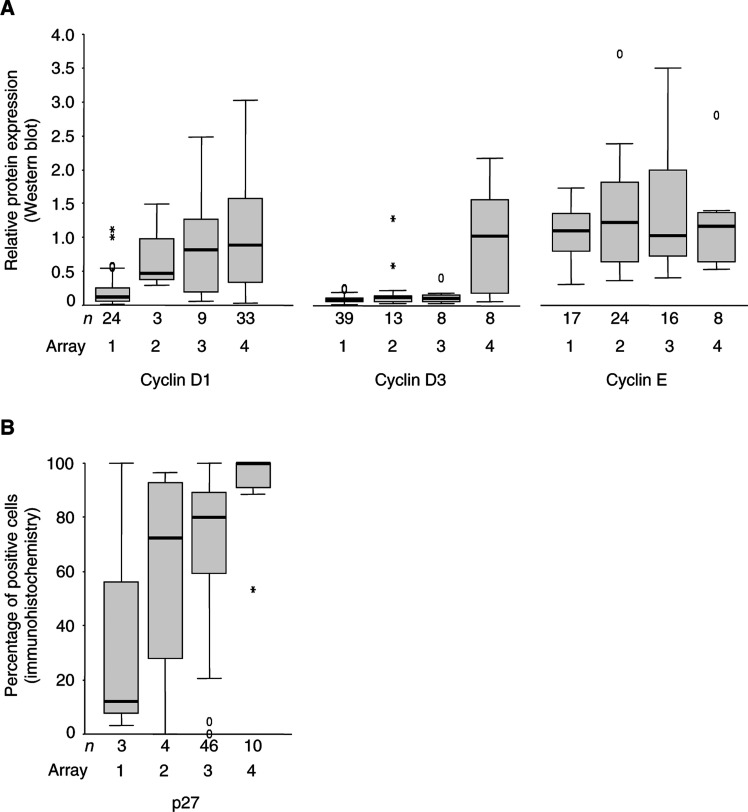
Comparison between TMA-based immunohistochemistry analysis of cyclin D1, D3, E, and p27 (1–4) with (**A**) Western-blot-based analysis (cyclin D1, D3, and E) or (**B**) immunohistochemistry of regular sections (p27).

. As illustrated in Figure 3A

, the cyclin D1 expression evaluated in the TMA correlated with the Western data (*P*<0.001). Similar correlation was obtained for the cyclin D3 staining (Figure 3A). The few cyclin D3 high RCCs, according to Western blot analysis, had high levels of cyclin D3 (scored as group 4) evaluated by the TMA (*P*<0.001). For cyclin E, no association was observed between the TMA evaluation and Western blot results (*P*=0.839, Figure 3A). Nevertheless, the TMA results correlated to the fraction of cyclin E positive cells evaluated by immunohistochemistry using regular sections (*P*<0.001, data not shown). The expression of p27 has been evaluated by immunohistochemistry using both the TMA technique and regular sections (Hedberg *et al*, 2002b) (the two methods corresponded significantly (*P*=0.009)), validating the results obtained from the TMA (Figure 3B).

### Conventional RCC

In conventional RCC, low cyclin D1 and p27 protein levels were associated with high nuclear grade and large tumour size (Table 2

**Table 2:** Association between cyclin D1/p27 expression and nuclear grade/tumour size in conventional RCC

	**Nuclear grade**				**Tumour size**		
	**1+2**	**3**	**4**	***P*****-value**	**Mean (cm)**	**Mean rank**	***P*****-value**
*Cyclin D1*							
Low	7	40	34	<0.001	8.7	102.1	<0.000
High	25	51	15		6.9	73.7	
							
*p27*							
Low	2	18	15	0.007	8.7	101.4	0.026
High	31	69	33		7.4	80.7	

). Expression of cyclin D1 and p27 were not linked to gender, age, or stage (data not shown). The protein contents of cyclin D3 and E were not associated with nuclear grade, tumour size, gender, age, stage, or survival (data not shown). Patients with cyclin D1^low^ tumours had an impaired survival compared with those having cyclin D1^high^ tumours (*P*=0.019, Figure 4A

**Figure 4:**
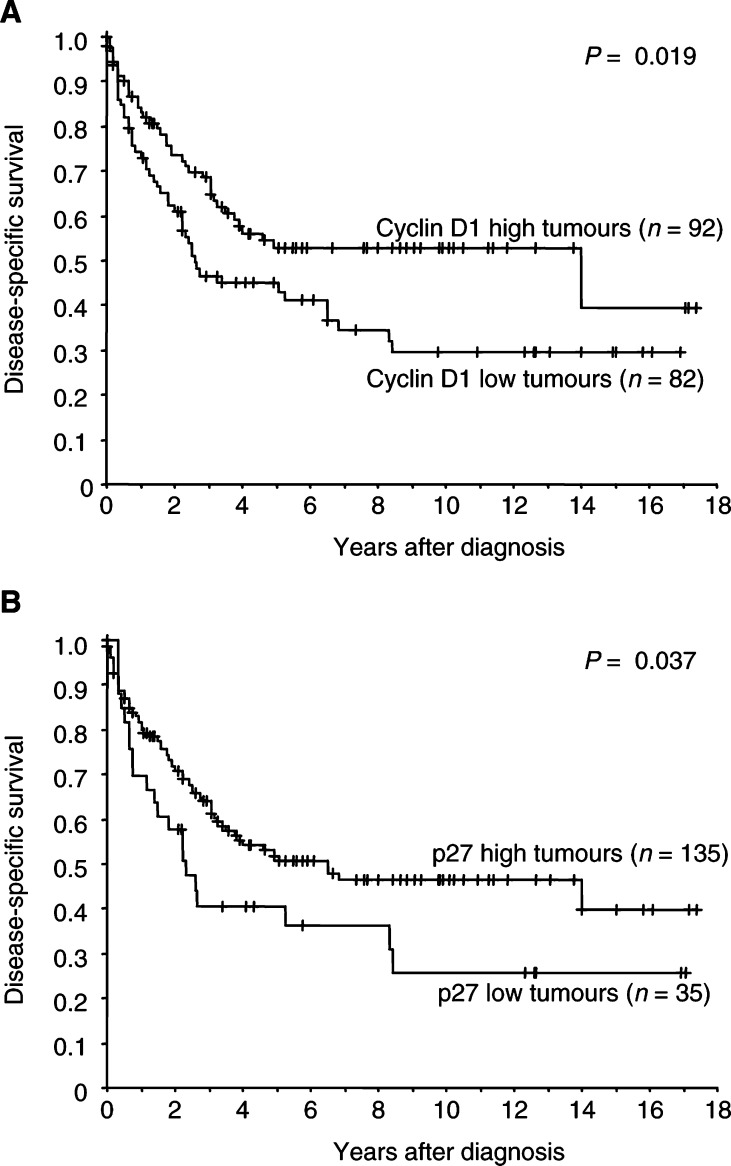
Kaplan–Meier survival curves for patients with conventional RCC: (**A**) cyclin D1 (low *vs* high levels); (**B**) p27 (low *vs* high levels).

). In addition, low levels of p27 were associated with a poor prognosis (*P*=0.037, Figure 4B). Multivariate analysis was performed in 155 conventional RCCs using cyclin D1, D3, E, p27, nuclear grade, and TNM as parameters (Table 3

**Table 3:** Multivariate Cox analysis of 155 conventional RCCs (77 events)

		**95% CI**		
**Variable**	**Exp (B)**	**Lower**	**Upper**	***P*****-value**
Cyclin D1 (low *vs* high)	0.899	0.500	1.618	0.723
Cyclin D3 (low *vs* high)	1.079	0.583	1.996	0.808
Cyclin E (low *vs* high)	1.247	0.742	2.094	0.405
p27 (low *vs* high)	0.386	0.194	0.766	0.007
Nuclear grade (1+2 *vs* 3+4)	6.053	1.879	19.493	0.003
TNM (I–III *vs* IV)	9.331	5.515	15.786	<0.001

). Independent significant factors were nuclear grade, TNM, and p27.

In the G1/S regulatory defects in conventional RCCs, we observed that tumours with high protein contents of cyclin D1, D3, or E also had high levels of p27 (Table 4

**Table 4:** Illustration of the association between the protein contents of cyclin D1, D3, E, and p27 in conventional, papillary, and chromophobe RCCs

	**Cyclin D1**			**Cyclin D3**			**Cyclin E**		
	**Low**	**High**	***P*****-value**	**Low**	**High**	***P*****-value**	**Low**	**High**	***P*****-value**
*Conventional*									
Cyclin D3									
Low	69	71	0.310						
High	11	18							
Cyclin E									
Low	66	52	0.001	106	10	<0.001			
High	16	39		34	19				
p27									
Low	34	1	<0.001	33	1	<0.010	30	5	0.009
High	44	90		103	28		84	50	
									
*Papillary*									
Cyclin D3									
Low	24	2	0.288						
High	2	1							
Cyclin E									
Low	15	3	0.529	16	2	1.000			
High	9	0		8	1				
p27									
Low	9	1	1.000	10	0	0.532	6	2	0.676
High	17	2		16	3		12	7	
									
*Chromophobe*									
Cyclin D3									
Low	10	2	0.396						
High	1	1							
Cyclin E									
Low	6	0	0.192	6	0	0.462			
High	4	3		5	2				
p27									
Low	9	0	0.027	9	0	0.110	6	2	0.021
High	2	3		3	2		0	5	

). Furthermore, cyclin E was significantly associated with cyclin D1 (*P*=0.001) and D3 (*P*<0.001), but there was no association between cyclin D1 and D3 protein levels. Figure 5A

**Figure 5:**
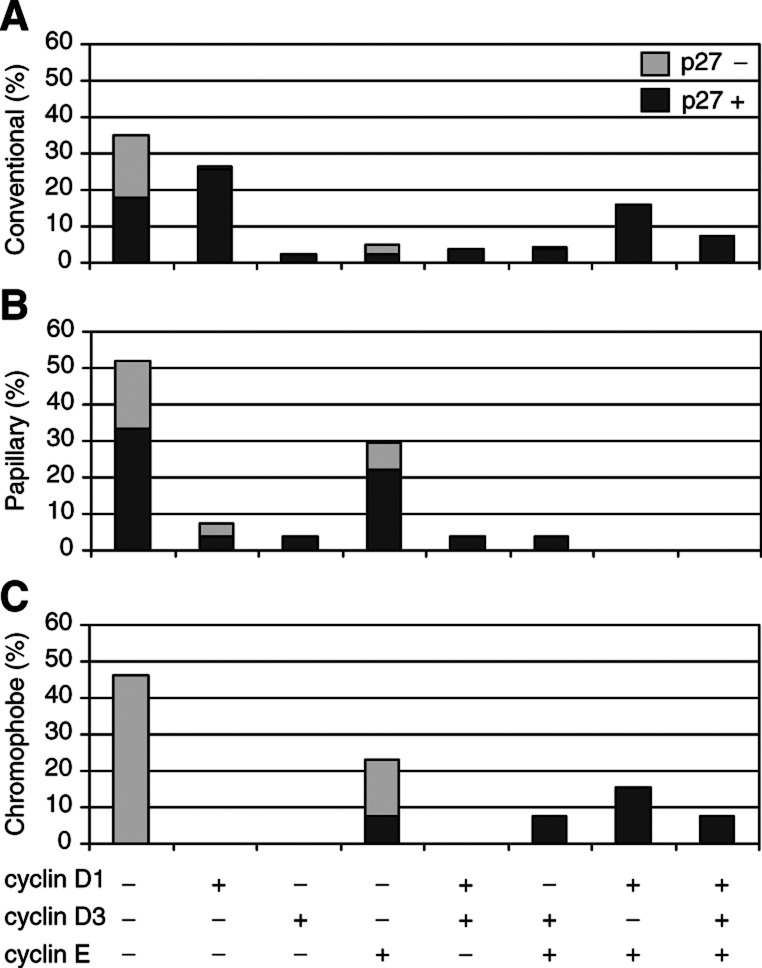
An illustration of the combinations of alterations in cell cycle regulatory proteins in (**A**) 163 conventional, (**B**) 27 papillary, and (**C**) 13 chromophobe RCC. The light grey bars represent tumours having low p27 levels, while the dark grey bars represent tumours with high p27 levels. The presence of low and high cyclin expression are indicated as − and +.

summarises the data from the analysed cell cycle regulatory proteins. The majority of the tumours that lacked p27 also lacked staining of all the three cyclins. Although these negative tumours were positive for VEGF, a protein served as a positive control for unspecific protein degradation (data not shown). Among the p27-positive tumours, cyclin D1 was the most frequently expressed cyclin either alone or in combination with cyclin E and/or cyclin D3.

### Papillary RCC

In papillary RCC, there was no association between cell cycle proteins and clinico-pathological parameters (data not shown). Furthermore, there was no correlation between the expressions of the different cell cycle proteins (Table 4). When the distribution of the cell cycle protein expression levels was analysed, two dominant expression patterns were observed (Figure 5B). The most common pattern (52% of the papillary RCC) showed low levels of all the three cyclins. The other main pattern, including 30% of the tumours, contained high p27 and cyclin E protein levels, but low levels, of cyclin D1 and D3. It should be noted that papillary RCC seldom expressed cyclin D1 or D3.

### Chromophobe RCC

In the rather few chromophobe RCCs (*n*=13), cyclin D1, D3, E, and p27 protein contents were not associated with any clinico-pathological parameters (data not shown). There was an association between p27 and cyclin D1 and E proteins (*P*=0.027, *P*=0.021, respectively) but not between p27 and cyclin D3 (*P*=0.110, Table 4). In addition, there was no correlation between the levels of cyclin D1, D3, and E, respectively. Chromophobe RCC showed different cell cycle regulatory protein expression patterns compared with conventional and papillary RCC (Figure 5C). The largest group of tumours (46%) had low levels of the three cyclins as well as p27. In the other tumours, cyclin E was highly expressed either alone or in combination with high cyclin D1 and/or D3 levels.

## DISCUSSION

In this study, the protein contents of cyclin D1, D3, E, and the cdk-inhibitor p27 were analysed in a large amount of RCC using the TMA technique and immunohistochemistry. Tissue microarray is a powerful method that enables analysis of large tumour materials treated in an identical manner, which reduces the slide-to-slide variability and the amount of reagents required. One disadvantage with this method is that rather small tissue areas are evaluated. This limits the amount of normal and tumour cells, which can be a problem when analysing different parameters in tumours with marked heterogeneity.

RCC is a disease with known heterogeneity regarding morphology and DNA ploidy (Ljungberg *et al*, 1996). However, it is still unclear whether the heterogeneity concerns cell cycle regulatory proteins. In this study, we found an agreement in staining and morphology between the two corresponding tissue cores in 95% of the tumours, suggesting a minor variation between the two biopsied areas. Our data correspond to the results obtained in a study on breast cancer, showing that the analysis of two tissue cores was comparable to the analysis of a whole tissue section in more than 95% of the cases (Camp *et al*, 2000).

By comparing the TMA results with the protein expression, we found that regular immunohistochemistry and Western blot validated the TMA technique. For p27, the TMA data correlated well to the regular immunohistochemical analysis and the staining of cyclin D1 and D3 on the TMA correlated to the corresponding Western blot analysis. In contrast, the cyclin E expression detected on the TMA disagreed with the Western blot analysis; however, the TMA results of cyclin E corresponded significantly to immunohistochemical stainings of regular tissue sections. These data suggest that the TMA technique was not the limiting factor. One reason for the disagreement might be the existence of isoforms of cyclin E, which potentially could affect the cyclin E evaluation. Several bands with different molecular weights were detected in the Western blot analysis (Hedberg *et al*, 2002b), and all bands were considered to represent the cyclin E protein (Keyomarsi *et al*, 1995). It is also possible that the antibody will not recognise all isoforms, due to the fixation procedure or due to other influences on the protein structure during the tissue processing.

The tumour specimens in the present study have been stored up to 18 years, but there was no indication that the staining efficiency was changed during the storage time. Our results agree with other studies on oestrogen receptor, progesterone receptor, and Her2/neu on breast cancer, which report that most paraffin-embedded materials up to an age of at least 68 years are still useful for immunohistochemical analysis (Camp *et al*, 2000). On the contrary, Larsson *et al* (1996) reported that tumours archived for many years lost the antigenicity for PCNA staining, suggesting that various antigens are affected differently by storage time. Nevertheless, the quality of the archived samples is an important aspect when designing TMAs. Our results suggest that up to 18-year old tumour samples can be used for immunohistochemical analysis of cyclin D1, D3, E, and p27.

We showed that the expression pattern of the evaluated cell cycle proteins varied between the different RCC types. These results agree with the finding that specific genetic aberrations characterise the different subtypes of RCC (Kovacs *et al*, 1997), although none of the genes for cyclin D1 (11q3), cyclin D3 (6p21), or cyclin E (19q12-13) are mapped to the chromosomes with characteristic abnormalities in RCC (Inaba *et al*, 1992; Demetrick *et al*, 1995; Kovacs *et al*, 1997).

In this study, we have shown that low levels of cyclin D1 and p27 correlated to poor prognosis in conventional RCC using univariate analysis, and that p27 remained significantly associated to survival in the multivariate analyses. The association between low cyclin D1 and poor prognosis in the univariate analysis agrees with the data reported for breast cancer (Gillet *et al*, 1996; Nielsen *et al*, 1997; Hwang *et al*, 2002; McKay *et al*, 2002), although an association between high cyclin D1 and poor prognosis has been reported in many malignancies (Åkervall *et al*, 1997; Gansauge *et al*, 1997).

In conclusion, we have analysed various cell cycle regulatory proteins in RCC and showed that the TMA technique is a reliable and useful technique for analysis of several proteins even in a heterogeneous tumour such as RCC. Aberrations in cyclin D1 and p27 were further associated with nuclear grade, tumour size, and patient survival. Interestingly, specific patterns of alterations in cell cycle regulators seemed to be characteristic for the different RCC types.
